# Neuroprotective Effect of a Nutritional Supplement Containing Spearmint Extract, Forskolin, Homotaurine and Group B Vitamins in a Mouse Model of Transient Ocular Hypertension

**DOI:** 10.3390/biomedicines11051478

**Published:** 2023-05-18

**Authors:** Andrea Satriano, Maria Luisa Laganà, Ester Licastro, Carlo Nucci, Giacinto Bagetta, Rossella Russo, Annagrazia Adornetto

**Affiliations:** 1 Preclinical and Translational Pharmacology, Glaucoma Unit, Department of Pharmacy, Health and Nutritional Sciences, University of Calabria, 87036 Rende, Italy; 2 Ophthalmology Unit, Department of Experimental Medicine, University of Rome Tor Vergata, 00173 Rome, Italy

**Keywords:** glaucoma, neuroprotection, dietary supplement, natural products, nutrients, apoptosis, retinal ischemia, retinal ganglion cells

## Abstract

Glaucoma is one of the most common sight-threatening eye disorders and one of the main causes of irreversible blindness worldwide. The current therapies focusing on reducing intraocular pressure (IOP) are often insufficient to prevent the progression of the disease, so the therapeutic management of glaucoma remains a challenge. The aim of this study was to evaluate the neuroprotective, IOP-lowering independent effects of a nutritional supplement containing forskolin, homotaurine, spearmint extract and vitamins of the B group in a model of acute glaucoma developed in mice. Glaucoma was induced in adult wild-type C57BL/6J mice by transient elevation of IOP. The dietary supplement, branded as Gangliomix^®^ (125 mg/kg/day), was administered by oral gavage for 17 days and ocular hypertension was induced on the 10th day of treatment. A histological analysis of the retinas was performed and RGC survival was evaluated with fluorogold labeling and Brn3a immunostaining on wholemount and retinal sections. Expression of alpha-spectrin, caspase-3, PARP-1 and GFAP was studied with western blotting or immunofluorescence. A significant increase in RGC survival was reported in the retina of mice treated with the dietary supplement as compared to vehicle-treated animals. The observed neuroprotection was associated with a calpain activity decrease, reduction in caspase-3 and PARP-1 activation, and prevention of GFAP upregulation. These effects were independent from the hypotensive effects of the supplement. Altogether, our data suggest that the dietary supplementation with forskolin, homotaurine, spearmint extract and vitamins of the B group supports RGC survival and may offer beneficial effects in glaucoma patients in combination with the currently used IOP-lowering therapy.

## 1. Introduction

Glaucoma is an age-related neurodegenerative disease characterized by progressive alterations of the optic nerve (ON) head and degeneration of retinal ganglion cells (RGCs) [1]. With more than 112 million people expected to be blind by 2040, it represents a world-leading cause of irreversible blindness [2]. Among the factors triggering the axonal degeneration and the apoptotic death of RGCs, elevated intraocular pressure (IOP) has been considered the main one and remains, to date, the only target for treatment [3]. However, the therapeutic management of glaucoma is still a challenge since lowering IOP is not always effective in preventing the progression of the disease [4] and, in a subset of glaucoma, defined as normal tension glaucoma (NTG), optic atrophy occurs under IOP values falling within the normal range [5]. Based on these observations, other unidentified IOP-unrelated factors are likely to be involved in triggering and sustaining the process ending with the loss of RGCs [6]. Indeed, several pathways have been shown to take part in the progression of the disease (i.e., inflammation, vascular alterations, autoimmunity, oxidative stress, excitotoxicity, alteration of neurotrophic factor support, mitochondria dysfunction, autophagy and others) [7,8,9,10,11,12] and therefore, the design of effective therapeutic strategies, supporting the currently available ocular hypotensive therapies, may rely on a combination of drugs with multiple targets, rather than on a single-target approach.

Experimental evidence suggests that nutritional supplementation with vitamins and natural compounds endowed with antioxidant, anti-inflammatory, neuroprotective or vasoactive properties is able to prevent or delay retinal degeneration [13,14] and has been proposed as an adjuvant to glaucoma treatments [15].

The active ingredients contained in the nutritional supplement tested in this study (Gangliomix^®^) include forskolin, homotaurine, a mix of vitamins (PP, B1, B2, B6, B12) and a polyphenolic fraction as part of the titrated spearmint extract. These components have been individually shown to exert beneficial effects in several in vivo and in vitro models of neurodegenerative diseases [15].

We have previously shown that the association of homotaurine, carnosine and forskolin (7 beta-acetoxy-8,13-epoxy-1 alpha, 6 beta, 9 alpha-trihydroxy-labd-14-ene-11-one) prevented RGC loss in an experimental model of acute glaucoma developed in rats [16]. The new supplement formulation is enriched with spearmint extract containing a polyphenolic fraction, which has been shown to be endowed with neuroprotective properties, ascribed to its antioxidant and anti-inflammatory activities, in vitro and in vivo [17,18,19,20,21].

The further addition of B-group vitamins is supported by compelling evidence suggesting that a deficit in nicotinamide adenine dinucleotide (NAD^+^) may drive ocular degeneration [22]. Indeed, recent studies have showed that, in the DBA/2J mouse model of glaucoma, a reduction in NAD^+^ and mitochondrial dysfunction are the first changes that can be observed in the retina. An NAD^+^ increase, either by oral administration of nicotinamide dietary supplementation or gene therapy, restored RGC function and provided a reversal in the disease metabolic profile of glaucomatous animals [22,23].

In the present study, we tested the efficacy of the dietary supplement containing forskolin, homotaurine, vitamins of the B group and spearmint extract, formulated with the commercial name of Gangliomix, in preventing RGC loss in a mouse model of acute glaucoma induced by transient ocular hypertension.

## 2. Materials and Methods

*Animals*: Male C57BL/6J mice (25–30 g) were purchased from Charles River (Lecco, Italy) and housed with a 12 h light–dark cycle with ad libitum access to food and water. The experimental protocol was approved by the Italian Ministry of Health (Rome, Italy; NIH license no. n.1100/2020-PR) and animal care and experimental procedures were carried out in accordance with the guidelines of the Italian Ministry of Health (D.L. 26/2014), the European Communities Council Directive (2010/63/UE) and the ARVO Statement for the Use of Animals in Ophthalmic and Vision Research. All surgical procedures were performed under deep anesthesia and efforts were made to minimize the number of animals and their suffering according to the 3Rs principles for ethical use of animals in scientific research.

*Dietary Supplementation*: The diet supplement Gangliomix^®^ (marketed by Fidia Farmaceutici S.p.A. Abano Terme, Italy) con was solubilized at the time of treatment in a 10% sucrose solution in PBS. The dose (125 mg/kg) was chosen based on the conversion of the doses used in humans (10 mg/kg, once or twice per day), considering the difference between species (Reagan-Shaw et al., 2008 [24]). The chosen dose contains the following amount of active ingredients: 19.3 mg/kg of dry extract of Coleus forskohlii titrated at 10% in forskolin, 14.5 mg/kg of homotaurine, 86.7 mg/kg of a dry extract of spearmint containing 20.9 mg/kg of total polyphenols and 12.6 mg/kg of rosmarinic acid, 2.7 mg/kg of vitamin PP, 0.4 mg/kg of vitamin B2, 0.4 mg/kg of vitamin B6, 0.3 mg/kg of vitamin B1 and 0.5 mg/kg of vitamin B12. A volume of 200 microliters of solution containing Gangliomix or a vehicle was administered with a plastic needle (20 gauge × 30 mm) for oral gavage (cod. FTP-20-30-50, 2Biological Instruments, Besozzo, Italy). Mice were gavaged every 24 h for 10 days before the induction of acute ocular hypertension and for the following 7 days.

*Transient Ocular Hypertension*: The transient increase in IOP was performed according to a method previously described [25]. Animals were deeply anesthetized with an intraperitoneal (i.p.) injection of a mixture of Xylazine (Sedaxilan 2%^®^, Dechra Veterinary Product, Torino, Italy) and Tiletamine-Zolazepam (Zoletil^®^, Virbac Srl, Milan, Italy) and laid on a heating pad to maintain the body temperature at 37 °C. Topical anesthesia was induced by 0.4% oxibuprocaine eye drops (Novesina, Novatis Farma, Italy). A 30-gauge infusion needle, connected to a 500 mL bottle of sterile saline, was inserted in the anterior chamber of the right eye, and the saline container was elevated to rise IOP above 90 mmHg for 60 min. Retinal ischemia was confirmed by whitening of the optic fundus. For each animal, the left eye was used as a control.

Body temperature was monitored before and after ischemia, and animals with values lower than 35.5 °C were excluded from the study. Animals were sacrificed at the indicated time points by cervical dislocation and retinas were quickly dissected and processed for the further analysis.

*Intraocular pressure recording*: IOP was monitored at the beginning and at the end of the ischemia, and after 1 h of reperfusion using a tonometer (Icare Lab/Tonolab, Italy). Corneal analgesia was achieved using topical oxibuprocaine 0.4% eye drops (Novesina, Novartis Farma, Italy); eyelids were gently retracted, and a tonometer probe was pointed at the central cornea. Each recorded value was the average of three repeated measurements.

*Retrograde labeling of RGCs*: To evaluate RGC loss, cells were labeled by injection of the fluorescent tracer FluoroGold (Fluka, Sigma-Aldrich, Milan, Italy) into the superior colliculus [26]. Spatial reference for the injection was provided by the standard stereotaxic coordinate system based on cranial landmarks (Paxinos and Watson, 2013 [27]). Four days after the ischemic insult, mice were anaesthetized and immobilized in a stereotaxic device (Kopf 900, Analytical Control, Milan, Italy), the skull was exposed and 2 μL of 2% Fluoro-Gold solution (Fluka, Sigma-Aldrich, Milan, Italy) was injected on both sides of the skull 4 mm posterior to the bregma, 1 mm lateral to the sagittal suture and 4 mm deep from the bone surface using a Hamilton syringe with a 33-gauge needle (Reno, NV, USA). The skin was sutured, and an antibiotic ointment was applied (0.3% tobramycin; Alcon, Milan, Italy). Seven days after ischemia (three days following FluoroGold injection), animals were sacrificed and eyeballs enucleated and fixed for 30 min in 4% paraformaldehyde (PFA). The anterior segment of the eye was removed and the posterior eye cup additionally fixed in PFA for 1 h. The isolated retinas were divided into four quadrants (nasal, temporal, upper and lower) and mounted on slides using a Vectashield medium (Vector Laboratories, DBA, Milan, Italy). Twenty images per retina (two from the peripheral, two from the middle and one from the central retina for each quadrant) were acquired using a confocal microscope (FV300, Olympus Corporation, Tokyo, Japan) and subjected to a cell count by a blind investigator. The total number of labeled cells in the ischemic eye (right) was compared to the contralateral eye (left) and expressed as a percentage of RGC loss.

*Preparation of flat-mount retina*: Enucleated eyes were fixed in 4% PFA for 30 min. The anterior chamber of the eye was removed and the posterior chamber post-fixed in 4% PFA for 1 h. Retinas were dissected out, divided into four quadrants and mounted on a SuperFrost slide. Tissue was incubated with a specific anti-GFAP [28,29] antibody at 4 °C overnight (Merck-Millipore, Burlington, MA, USA, dilution 1:300) and then with donkey anti-mouse Alexa Fluor 594 (dilution 1:1250) for 1 h at room temperature. Specimens were mounted with a Fluoroshield mounting medium containing 4′,6-diamidino-2-phenylindole (DAPI) (cod. AB104139, Abcam, Cambridge, UK) for nucleic acid staining. The fluorescent signal was analyzed using an FV3000 confocal laser scanning microscope (Olympus Corporation, Tokyo, Japan).

*Immunohistochemistry*: Following the induction of ocular hypertension, mice were sacrificed and the eyes were quickly removed and fixed in 4% PAF for 24 h. Tissue was subjected to dehydration in 75% ethanol for 48 h at 4 °C, subsequently to progressively increasing concentrations of ethanol (95%, 100%) and finally immersed in xylene for 1 h at room temperature. Next, 7 μm thick sections were obtained from the eye embedded in paraffin, through the use of a microtome, and mounted on a SuperFrost slide. Sections were dewaxed by immersion in pure xylene, increasing concentrations of ethanol and water. Slides were stained in a hematoxylin solution for 15 min at room temperature and, after washing, in a 1% eosin solution for 10 s, mounted with transparent sealant and analyzed using an optical microscope BX41 (Olympus Corporation, Tokyo, Japan). The number of RGCs was counted per 100 μm within the ganglion cell layer (GCL) in five randomly selected sections, compared with the contralateral eye and expressed as a percentage of cells in the GCL.

All images were acquired from the mid-peripheral area of the retinas. Measurement of retinal thickness was made with the aid of the ocular micrometer (Olympus Corporation, Tokyo, Japan) calibrated on the 20× microscope objective.

*Protein extraction and western blotting*: Retinas were lysed in an ice-cold RIPA buffer (50 mM Tris-HCl (pH 8), 150 mM NaCl, 1 mM ethylenediaminetetraacetic acid, 0.1% sodium dodecyl sulfate, 1% IGEPAL, 0.5% Sodium deoxycholate) containing protease (cod. P8349; Sigma-Aldrich, Milan, Italy) and phosphatase inhibitor (cod. P5726; Sigma-Aldrich, Milan, Italy) cocktails. Lysates were centrifuged for 15 min, 10,000× *g* at 4 °C, and supernatants were assayed for protein content with the Bio-Rad DC Protein Assay Kit (Bio-Rad Laboratories, Milan, Italy). An equal amount of total proteins (10 μg) was separated by sodium dodecyl sulfate-polyacrylamide gel electrophoresis (SDS-PAGE), transferred onto PVDF membranes (Immobilon-P, Sigma-Aldrich, Milan, Italy) and blocked with 5% non-fat milk in Tris-buffered saline containing 0.05% Tween 20 for 1 h at room temperature. Primary antibodies were incubated overnight at 4 °C. The following primary antibodies and dilutions were used: an anti-Caspasi-3 1:1000 dilution (Cell Signaling Technology, Beverly, MA, USA), anti-GFAP 1:1000 dilution (Merck-Millipore, Burlington, MA, USA), anti-spectrin 1:1000 dilution (Merck-Millipore, Burlington, MA, USA), anti-actin 1:1000 dilution (clone AC-40, Sigma-Aldrich, Milan, Italy) and anti-tubulin 1:20,000 (Sigma-Aldrich, Milan, Italy). Species-specific horseradish peroxidase conjugated goat immunoglobulin G (IgG; Pierce Biotechnology, Rockford, IL, USA) was used as secondary antibodies. Protein bands were visualized with a Western Blotting Luminol Reagent (Santa Cruz Biotechnology, Dallas, TX, USA) and the chemiluminescence signal was detected using X-ray films (Santa Cruz Biotechnology, Dallas, TX, USA). Autoradiographic films were scanned and digitalized at 600 dpi, and band quantification was performed using ImageJ software v.1.5.3 (NIH, Bethesda, MD, USA).

*Immunofluorescence*: At the indicated time points, the animals were deeply anesthetized and eyeballs were fixed in 4% paraformaldehyde at 4 °C for 1 h, cryopreserved in 15% sucrose overnight and then in 30% sucrose for 1 week [30]. Specimens were frozen in an Optimal Cutting Temperature compound (Tissue-Tek^®^, Sakura Finetek Europe, Alphen aan den Rijn, The Netherlands), and 14 μm cryostat sections were cut, mounted onto Superfrost Plus glass slides (Thermo Fisher Scientific, Waltham, MA, USA) and stored at −80 °C until use. For cellular and subcellular localization of specific antigens, retinal sections were thawed, air-dried, post-fixed in 4% paraformaldehyde for 15 min and washed in 0.1 M PBS (pH 7.4). Sections were permeabilized with 0.3% Triton-X100 (Sigma-Aldrich, Milan, Italy) for 1 h and blocked with a 10% donkey serum (Sigma-Aldrich, Milan, Italy) at room temperature for 1 h. Sections were incubated overnight at 4 °C with an anti-Brn3a [31,32] primary antibody (dilution 1:50, Santa Cruz Biotechnology, Dallas, TX, USA), washed three times and then incubated with donkey anti-mouse Alexa Fluor 488, 1:500 (Molecular Probes, Eugene, OR, USA) for 1 h at room temperature. After washing with a PBS solution, slides were mounted with Vectashield mounting media containing DAPI (Vector Laboratories, Burlingame, CA, USA) and fixed with a coverslip. Single scan image acquisition was performed using a confocal microscope (FV3000, Olympus Corporation, Tokyo, Japan).

*Statistical analysis*: Data are expressed as mean ± SEM of three to six independent experiments and were evaluated statistically for differences with an ANOVA followed by a Tukey–Kramer test for multiple comparisons. Where indicated, a Student *t*-test was used to evaluate differences between two means. A value of *p* less than 0.05 was considered to be significant.

## 3. Results

### 3.1. Oral Administration of Gangliomix Prevented RGC Loss Induced by Transient Ocular Hypertension

Induction of transient ocular hypertension is associated with a significant loss of RGCs and alteration of the retinal laminar structure [28,33].

To evaluate the neuroprotective effect of Gangliomix in our experimental model, the dietary supplement was administered by oral gavage at the dosage of 125/mg/kg/die. The posological regimen was chosen based on the human equivalent dose (10 mg/kg/die) and on previous studies showing efficacy of oral supplementation with Gangliomix in a mouse model of ON damage [34] and ocular hypertension [35]. The dietary supplement was administered once a day for 17 days and ocular hypertension was induced on the 10th day.

**Figure 1:**
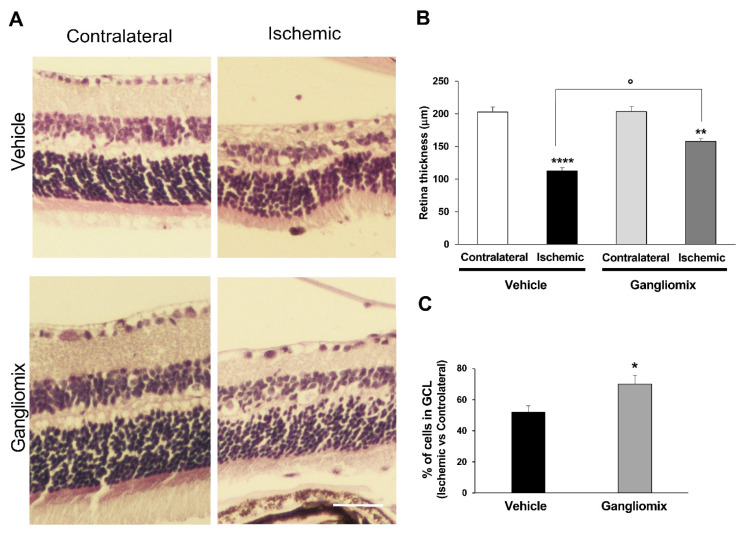
Gangliomix prevents the reduction of cellularity in GCL and partially preserves retinal thickness following ischemia/reperfusion injury. (**A**) Representative images from mid-peripheral retina at 7 days of reperfusion stained with hematoxylin and eosin (H&E). Mice were subjected to oral administration of Gangliomix 125 mg/kg or vehicle for 10 days before retinal ischemia and for the following 7 days (scale bar = 50 μm). (**B**) Histograms show the result of retinal thickness. Measurement of retinal thickness was made with the aid of ocular micrometer calibrated on the 20× microscope objective (4–5 randomly selected sections for each group; **** *p* < 0.0001 Ischemic versus Contralateral + Vehicle; ** *p* < 0.01 Ischemic + Gangliomix versus Contralateral + Gangliomix; and ° *p* < 0.05 Ischemic + Gangliomix versus Ischemic). (**C**) Histograms show the number of cells in GCL layer versus contralateral reported as mean ± SEM (3–5 independent experiments for each group; * *p* < 0.05 versus vehicle-treated ischemic retinas).

The histochemical analysis of H&E-stained retinas showed that dietary supplementation with Gangliomix prevented the changes in the retinal laminar structure induced by the hyperbaric damage (Figure 1). After 7 days of reperfusion, the retinal thickness (Figure 1A,B) and the GCL density (Figure 1A,C) were partially preserved in mice treated with the supplement and subjected to transient ocular hypertension as compared to vehicle-treated mice.

**Figure 2:**
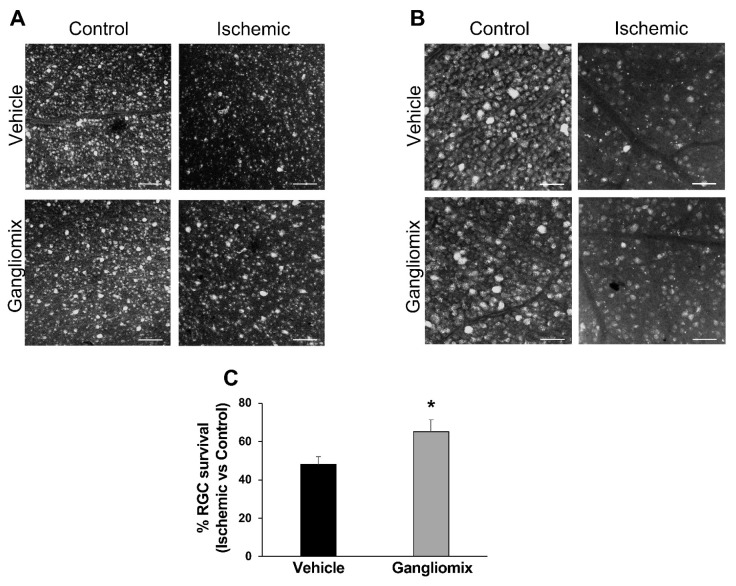
Oral administration of Gangliomix reduces RGC loss induced by transient ocular hypertension. (**A**,**B**) Representative fluorescent photomicrograph from mid-peripheral retina of whole-mount control and ischemic retinas from vehicle- and Gangliomix-treated mice. Oral administration of Gangliomix 125 mg/kg significantly increased the percentage of FluoroGold (FG)-labeled RGCs in the ischemic retinas as compared with vehicle-treated mice. Images are representative of four independent experiments ((**A**), scale bar = 100 μm; (**B**) scale bar = 50 μm)). (**C**) Histograms show the result of RGC count. Twenty images per retina were acquired, and FG-labeled cells were counted. The total number of labeled cells in the ischemic retina was compared with the contralateral eye and expressed as percentage of RGC survival. Results are reported as mean ± SEM of four independent experiments (* *p* < 0.05 versus vehicle-treated ischemic retinas).

As shown in Figure 2, Gangliomix administration significantly prevented RGC loss induced by transient ocular hypertension. Indeed, the percentage of Fluorogold-labeled cells in flat mount ischemic retinas as compared to contralateral non-ischemic tissue was significantly higher in mice under dietary supplementation as compared to vehicle-treated mice (Gangliomix = 65 ± 6.3 vs. Vehicle = 48 ± 4.2).

**Figure 3:**
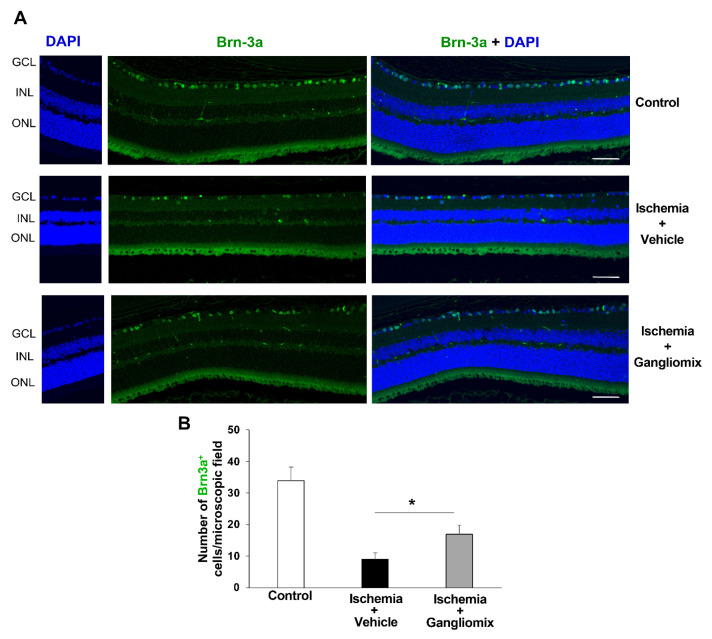
Decline of Brn3a-positive cells observed in vehicle-treated retinas following ischemia/reperfusion injury is prevented by the dietary supplementation with Gangliomix. (**A**) Confocal images of retina sections from mid-peripheral labeled with anti-Brn3a (green), an RGC-specific marker. The number of Brn3a-immunolabeled RGCs decreased in the ischemic retina as compared to control. Brn3a-immunopositive cells were partially preserved in the ischemic retinas from mice treated with Gangliomix. Nuclei were counterstained with DAPI (blue) (GCL, ganglion cell layer retinal; INL, inner nuclear layer; ONL, outer nuclear layer) (20× objective with a 2.5× zoom). Non-specific background signal in the ONL is due to tissue autofluorescence. (**B**) Histograms show the number of cells positive for Brn3a per microscopic field reported as mean ± SEM (3–5 independent experiments for each group, * *p* < 0.05 versus vehicle-treated ischemic retinas). Scale bar = 50 μm.

Furthermore, the immunolabeling of retina sections for Brn3a (brain-specific homeobox/POU domain protein 3), a marker of adult RGCs [31,32], showed that the decline in Brn3a-positive cells observed in the vehicle-treated retinas was prevented by the dietary supplementation (Figure 3).

**Figure 4:**
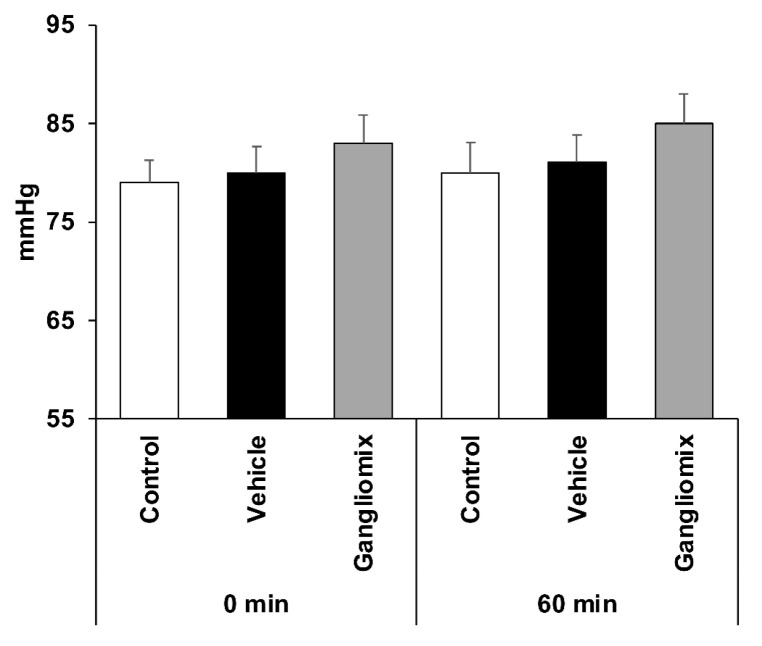
No changes in IOP values are reported in mice treated with Gangliomix. Histograms report IOP values recorded right after the induction of ocular hypertension and just before ending the procedure. No significant changes in IOP values are reported in Gangliomix-treated eyes compared to control and vehicle-treated. Results are reported as mean ± SEM of four independent experiments.

IOP values were unchanged in Gangliomix-treated mice as compared to control and vehicle-treated ones (Figure 4), suggesting that the neuroprotective effect of Gangliomix was IOP-independent.

### 3.2. Dietary Supplementation with Gangliomix Prevented the Activation of Cell Death Pathways Induced by Transient Elevation of Intraocular Pressure

Activation of calpain, Ca^2+^-dependent enzymes involved in cell death [36], has been reported following retina ischemic insult reaching a peak after 1 h of reperfusion [16,28], and this event has been linked to the extent of RGC damage [37].

To evaluate calpain activation, we monitored calpain-specific, 150/145 kDa alfa-spectrin breakdown products (SBDPs) with immunoblotting [38].

**Figure 5:**
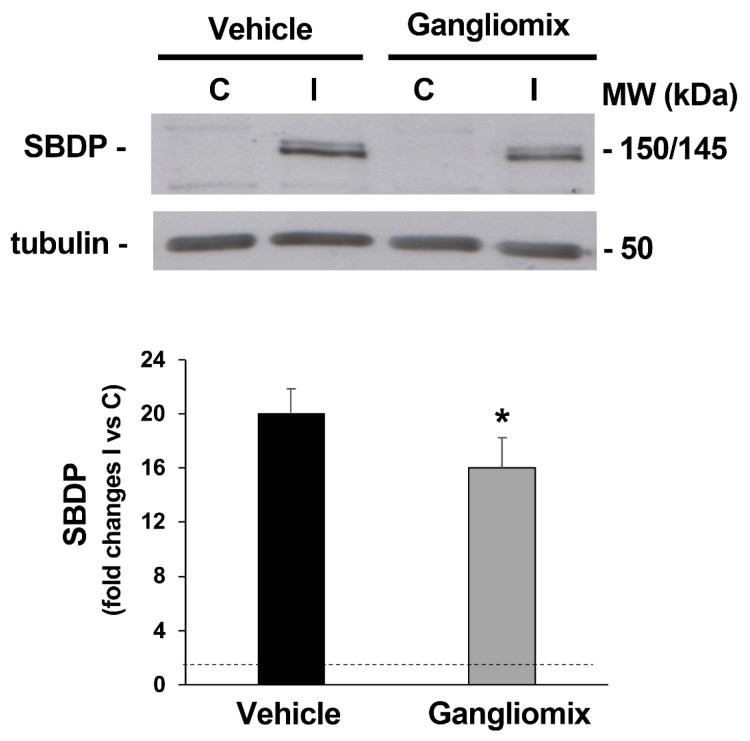
Gangliomix prevents calpain activation following retinal ischemia/reperfusion. Immunoblotting shows the typical increase in calpain-specific, 150/145 kDa alfa-spectrin breakdown products (SBDPs) observed in the ischemic retina after 1 h of reperfusion. Oral supplementation with Gangliomix significantly reduced calpain activation as shown by the reduced intensity of the 150/145 kDa SBDP bands. A representative immunoblot from four independent experiments is shown. Histograms show the result (expressed as mean ± SEM of four experiments) of the densitometric analysis of the autoradiographic bands relative to 150/145 SBDPs normalized to the value of tubulin and compared to the contralateral eye (* *p* < 0.05 versus vehicle-treated ischemic retinas) (C: control non-ischemic retina; I: ischemic retina; MW: molecular weight).

As shown in Figure 5, in the ischemic retina of mice fed with the vehicle, as compared to the control, a significant accumulation in the 145/150 SBDPs was reported; a significant reduction in SBDP accumulation was detected in the ischemic retinas of mice subjected to dietary supplementation as compared to those treated with the vehicle alone (Figure 5), suggesting that Gangliomix prevented the activation of calpains in our experimental model.

**Figure 6:**
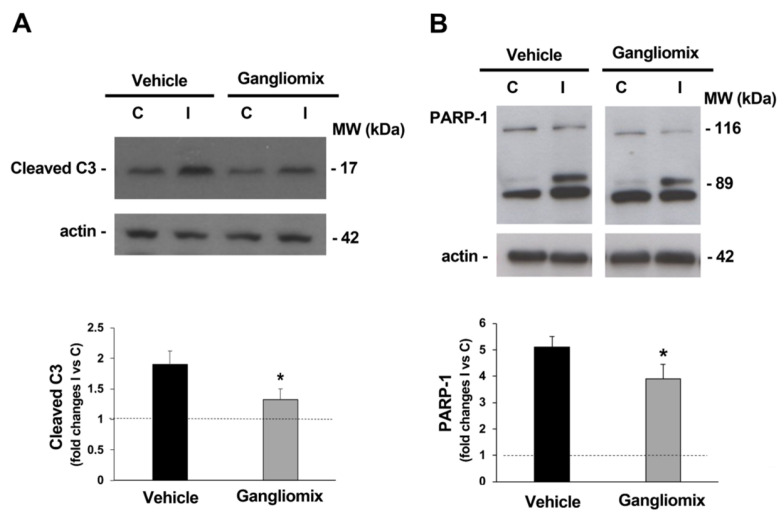
Oral supplementation with Gangliomix prevents the upregulation of apoptotic-related proteins induced by retina ischemia/reperfusion. Treatment with Gangliomix prevents caspase-3 activation (**A**) and caspase-3-mediated PARP-1 cleavage (**B**) following 24 h of reperfusion. Immunoblotting shows the typical increase in the apoptotic markers appearing in the ischemic retinas at 24 h of reperfusion and the effects of the dietary supplementation with Gangliomix. A representative immunoblot from four independent experiments is shown. Histograms show the results (expressed as mean ± SEM of four independent experiments) of the densitometric analysis of the autoradiographic bands normalized to the value of actin and compared to the contralateral eye (* *p* < 0.05 versus vehicle-treated ischemic retinas, MW: molecular weight).

To evaluate if the dietary supplement was able to affect the expression of proteins involved in the apoptotic pathway, the activation of caspase-3, a cysteine-aspartic acid protease playing a central role in the execution-phase of apoptosis, was studied [39,40]. As shown in Figure 6, accumulation of the caspase-3 active fragment was reported in the ischemic retina from vehicle-treated mice at 24 h of reperfusion (Figure 6A) together with the accumulation of the 89 kD caspase-3-mediated poly (ADP-ribose) polymerase (PARP) cleavage fragment (Figure 6B) [41]. Both the activation of caspase-3 and the accumulation of caspase-3 dependent PARP fragments were prevented in the ischemic retina from mice who had undergone dietary supplementation with Gangliomix (Figure 6).

### 3.3. Dietary Supplementation with Gangliomix Prevented Astrocyte Activation Induced by Ocular Hypertension

**Figure 7:**
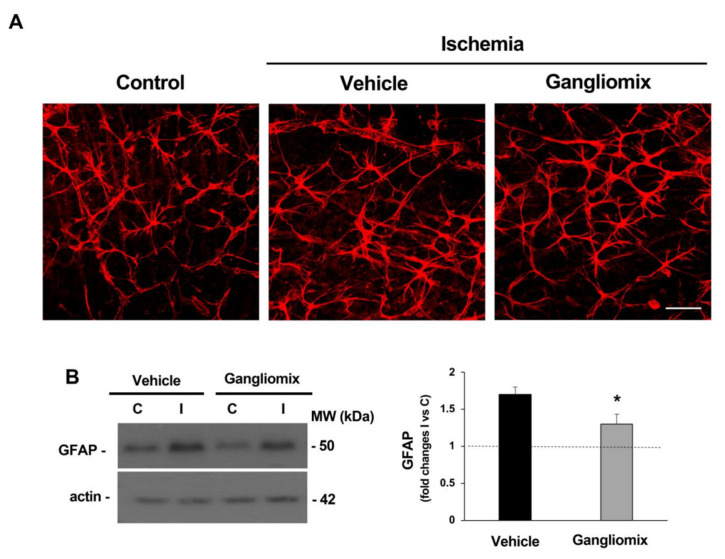
Gangliomix supplementation decreases the upregulation of GFAP induced by retinal ischemia/reperfusion. (**A**). Representative immunofluorescence experiments on whole-mount retina showing the effect of oral administration of Gangliomix 125 mg/kg on GFAP immunoreactivity. Reduced GFAP expression is evident in the retina treated with Gangliomix as compared to vehicle-treated. Scale bar = 50 μm. (**B**). Representative western blotting showing the expression levels of GFAP in the control and ischemic retina homogenates from vehicle- and Gangliomix-treated mice at 7 days of reperfusion. Histograms report the results (expressed as mean ± SEM of four independent experiments) of the densitometric analysis of the autoradiographic bands relative to GFAP, normalized to the value of actin and compared to the contralateral eye (* *p* < 0.05 versus vehicle-treated ischemic retinas) (C: control non-ischemic retina; I: ischemia/reperfusion; MW: molecular weight).

Astrogliosis, as part of a complex inflammatory response to damage, has been observed in the retina during the early stages of reperfusion [42,43,44]. In particular, activation in response to IOP elevation includes a retraction of astrocytic processes and an upregulation of structural proteins such as glial fibrillary acidic protein (GFAP) [28,29]. Here, we observed that astrocyte activation and the corresponding increase in GFAP expression were mitigated by dietary supplementation with Gangliomix (Figure 7), suggesting that anti-inflammatory effects take part in the observed neuroprotective effect.

## 4. Discussion

Glaucoma is a multifactorial optic neuropathy leading to the progressive loss of the visual field due to the death of RGCs [45]. The disease not only affects the retina and the ON but also other areas of the visual pathway, such as the lateral geniculate bodies and the visual cortex [46,47]. The mechanisms triggering the apoptotic cascade in glaucoma are similar to those of other central neurodegenerative disorders, such as Alzheimer’s disease [48,49], and engage several pathways. Inflammation, oxidative stress, mitochondrial dysfunction, excitotoxicity, ischemia, alteration of trophic factor transport and autophagy dysfunction contribute to triggering and sustaining a vicious cycle, creating a hostile environment for RGC survival and a condition of chronic neuroinflammation [7,8,9,10,11,12].

The recognized neurodegenerative nature of glaucoma is the basis for the identification of neuroprotective strategies, adjuvant of the therapy lowering IOP, which remains the only known modifiable risk factor [50,51]. Neuroprotection aims at preventing or delaying RGC death while sustaining its survival, an intervention that has been defined as neuroenhancement [52,53].

With this end, the present study assessed the neuroprotective potential of the oral intake of a dietary supplement, branded as Gangliomix^®^, containing forskolin, homotaurine, spearmint extract and vitamins of the B group, in a mouse model of acute glaucoma induced by transient elevation of IOP. Following the dietary supplementation, we observed an increased survival of RGCs and preservation of Brn3a immunostaining, downregulation of proteins linked to the apoptotic and necrotic cell death process and reduced astrogliosis.

Our results confirm the neuroprotective efficacy of Gangliomix in animal models of RGC injuries. A previous study by Locri and colleagues (2019) in a mouse model of optic nerve crush (ONC) showed that treatment with Gangliomix promotes RGC survival and modulates inflammatory and apoptotic processes [34]. Similarly, Cammalleri et al., 2020, using a model of hypertensive glaucoma, reported that dietary supplementation with Gangliomix prevents RGC death, preserves RGC function and modulates inflammatory and apoptotic pathways [35].

Among the constituents of Gangliomix, the spearmint extract is rich in polyphenolic compounds, mainly rosmarinic acid [54], which is known for its anti-inflammatory, anti-oxidant and anti-aging effects, as well as for inhibiting cell proliferation and migration [19,55]. The neuroprotective activity of flavonoids and catechins, a group of polyphenolic compounds extracted from several sources (i.e., Ginkgo Biloba, green tea), has been shown to exert protective effects on injured RGCs through reduced inflammation and oxidative stress in different models of glaucoma [15]. More importantly, a systematic analysis of the clinical data suggests that flavonoid intake can help preserve the visual field in patients with ocular hypertension or glaucoma without modifying IOP values [56,57,58].

Forskolin and homotaurine, endowed with well-known neuroprotective properties, are among the bioactive molecules of the dietary supplement. Forskolin, a natural diterpenic adenylate cyclase activator extracted from the roots of Coleus Forskohlii, has been shown to support RGC survival by exerting neurotrophin-like activity [59,60] and by decreasing IOP through the reduction in aqueous humor production [61,62,63,64,65]. Homotaurine, introduced in the clinic under the name of tramiprosate [66], is a natural aminosulfonate compound with neuromodulatory and neuroprotective properties as reported in primary retinal cell cultures exposed to glutamate [67] and in several in vitro and in vivo models mimicking neurodegenerative disorders [68,69].

In a previous study, our group tested the effects of the intravitreal administration of forskolin, homotaurine and carnosine in a rat model of retinal ischemia/reperfusion induced by transient elevation of IOP [16]. Forskolin partially sustained RGC survival and this effect was implemented by the simultaneous administration of homotaurine and carnosine [16]. Under these experimental conditions, neuroprotection was independent from the previously demonstrated hypotensive action of forskolin [61,62,63,64,65] and associated with the upregulation of the pro-survival phosphoinositide-3-kinase/protein kinase B (PI3K/Akt) pathway. Here, although the route and timing of administration and, consequently, the bioavailability of each compound are different, we can hypothesize that upregulation of the PI3K/Akt pathway may also be part of the neuroprotective mechanisms afforded by Gangliomix.

Excitotoxicity is an important contributor of the neurodegenerative process induced by retinal ischemia/reperfusion [10]. In particular, calcium overload triggered by the overstimulation of NMDA receptors leads to the activation of calpain, calcium-dependent proteases, involved in the process of RGC death [10,28,70]. Indeed, pharmacological inhibition of calpain prevents cell loss in the ganglion cell layer induced by acute ocular hypertension [71,72].

Previous studies have shown that forskolin alone or in combination with homotaurine and carnosine is able to limit excitotoxic damage and calpain activity [16]. Here, we reported similar results showing that the oral supplementation with Gangliomix is associated with a reduced index of excitotoxic toxicity. However, under the present condition, we cannot ascribe this effect to a single bioactive ingredient of Gangliomix and it can be hypothesized that the final effects are due to more than one mechanism.

Gangliomix supplementation results in a decreased activation of caspase-dependent apoptosis shown by the decreased activity of the effector caspase-3 and the reduction in the caspase-3-mediated PARP cleavage. This is in agreement with previous findings showing that treatment with Gangliomix is associated with a decreased activation of the apoptotic cascade, measured by the improved ratio of Bax/Bcl2 and the reduction in caspase-3 expression in a mouse model of optic nerve crush [34] and following the induction of ocular hypertension by methylcellulose (MCE) injection [35].

Among the B vitamins contained in Gangliomix, vitamin B3 (niacin), the precursor of nicotinamide adenine dinucleotide (NAD^+^) and NAD phosphate (NADP^+^), plays a key role in neuronal development and survival [73]. An aging-dependent decline in NAD+ retinal levels has been described [74] and recent studies by Williams and collaborators in aged DBA/2J mice have shown that a diet supplemented with nicotinamide (the amide form of vitamin B3) rescues the glaucomatous phenotype of these mice by reducing mitochondrial vulnerability and protecting RGCs from degeneration [22]. Interestingly, a significantly lower concentration of the amide form of vitamin B3 (nicotinamide) has been reported in a cohort of POAG as compared to controls [75]; in a randomized clinical trial enrolling glaucoma patients under IOP-lowering therapy, oral supplementation with nicotinamide improved inner retinal function [76]. Therefore, the neuroprotective effects sustained by a dietary supplementation with Gangliomix may also rely on the improvement of NAD+ biosynthesis.

A more in-depth study, dissecting single mechanisms involved in the neuroprotection afforded by a dietary supplementation with Gangliomix, appears to be biased by several factors in terms of research, development and translation to glaucoma patients.

Although each component of the supplement is able to act on specific target-modulating key pathways involved in RGC death (i.e., inflammation, oxidative stress, mitochondrial dysfunction, apoptosis), it is conceivable that their combination may provide synergic and additive effects that cannot be attributed to a single ingredient. Furthermore, a complete study should consider not only direct effects of the dietary supplementation on the retinal homeostasis, either under physiological or pathological states, but also indirect systemic effects (i.e., immunomodulation, changes of the metabolic state, systemic antioxidant capacity).

It must also be taken into account that each animal model of glaucoma has limitation since it only recapitulates some features of the human pathology and, therefore, the transferability of the experimental data needs caution.

In conclusion, our data strengthen the neuroprotective activity of Gangliomix and suggest that this dietary supplement may offer beneficial effects in glaucoma patients in combination with the current IOP-lowering therapy.

## Data Availability

The data presented in this study are available on request from the corresponding author.
